# Transcriptomic responses to grazing reveal the metabolic pathway leading to the biosynthesis of domoic acid and highlight different defense strategies in diatoms

**DOI:** 10.1186/s12867-019-0124-0

**Published:** 2019-02-26

**Authors:** Sara Harðardóttir, Sylke Wohlrab, Ditte Marie Hjort, Bernd Krock, Torkel Gissel Nielsen, Uwe John, Nina Lundholm

**Affiliations:** 1 Natural History Museum of Denmark, University of Copenhagen, Øster Farimagsgade 5, 1353 Copenhagen K, Denmark; 2 Alfred Wegener Institute, Helmholtz Centre for Polar and Marine Research, Am Handelshafen 12, 27570 Bremerhaven, Germany; 3 Helmholtz Institute for Functional Marine Biodiversity, Ammerländer Heestraße 231, Oldenburg, Germany; 4 National Institute of Aquatic Resources, Technical University of Denmark, Building 201, Kemitorvet, Lyngby Campus, 2800 Kgs. Lyngby, Denmark

**Keywords:** *Pseudo-nitzschia*, *Fragilariopsis*, Grazer induced defense, Domoic acid, Gene expression, Methyl-erythritol phosphate metabolic pathway, Geranyl pyrophosphate, l-Glutamate, Proline

## Abstract

**Background:**

A major cause of phytoplankton mortality is predation by zooplankton. Strategies to avoid grazers have probably played a major role in the evolution of phytoplankton and impacted bloom dynamics and trophic energy transport. Certain species of the genus *Pseudo-nitzschia* produce the neurotoxin, domoic acid (DA), as a response to the presence of copepod grazers, suggesting that DA is a defense compound. The biosynthesis of DA comprises fusion of two precursors, a C10 isoprenoid geranyl pyrophosphate and l-glutamate. Geranyl pyrophosphate (GPP) may derive from the mevalonate isoprenoid (MEV) pathway in the cytosol or from the methyl-erythritol phosphate (MEP) pathway in the plastid. l-glutamate is suggested to derive from the citric acid cycle. *Fragilariopsis*, a phylogenetically related but nontoxic genus of diatoms, does not appear to possess a similar defense mechanism. We acquired information on genes involved in biosynthesis, precursor pathways and regulatory functions for DA production in the toxigenic *Pseudo-nitzschia seriata*, as well as genes involved in responses to grazers to resolve common responses for defense strategies in diatoms.

**Results:**

Several genes are expressed in cells of *Pseudo-nitzschia* when these are exposed to predator cues. No genes are expressed in *Fragilariopsis* when treated similarly, indicating that the two taxa have evolved different strategies to avoid predation. Genes involved in signal transduction indicate that *Pseudo-nitzschia* cells receive signals from copepods that transduce cascading molecular precursors leading to the formation of DA. Five out of seven genes in the MEP pathway for synthesis of GPP are upregulated, but none in the conventional MEV pathway. Five genes with known or suggested functions in later steps of DA formation are upregulated. We conclude that no gene regulation supports that l-glutamate derives from the citric acid cycle, and we suggest the proline metabolism to be a downstream precursor.

**Conclusions:**

*Pseudo-nitzschia* cells, but not *Fragilariopsis*, receive and respond to copepod cues. The cellular route for the C10 isoprenoid product for biosynthesis of DA arises from the MEP metabolic pathway and we suggest proline metabolism to be a downstream precursor for l-glutamate. We suggest 13 genes with unknown function to be involved in diatom responses to grazers.

**Electronic supplementary material:**

The online version of this article (10.1186/s12867-019-0124-0) contains supplementary material, which is available to authorized users.

## Background

Phytoplankton survival depends, among other factors, on the capability to defend the cells against grazing zooplankton. Predation is the main source of phytoplankton mortality [1] and diatoms have evolved a variety of strategies to reduce predation. Thick silicate frustules and spiny armors, formation of toxins, adaptable cell sizes and formation of long chains are examples of defense traits in diatoms [2]. Defense mechanisms induced by the presence of predators require cells to sense and recognize the predator from a distance. Diatoms exhibit complex signaling mechanisms that allow perception of environmental cues such as the presence of gametes of opposite sex and the presence of bacteria [3, 4]. Diatoms of the genera *Pseudo-nitzschia* and *Skeletonema* respond to predator cues from copepods: at least two *Pseudo-nitzschia* species by inducing toxin production, and *Skeletonema marinoi* by shortening the chain length [5–7]. The induced defense responses are both diatom specific and predator specific [5–7]. Thus two phylogenetically related diatoms, *Fragilariopsis cylindrus* and *Niztschia frigida*, do not induce formation of domoic acid (DA). *Pseudo-nitzschia seriata* elicits induced DA production in response to predator cues from herbivorous copepods, but when exposed to a copepod carnivore, a response is not elicited [8]. Predator cues as referred to in this paper are info-chemical signals that mediate interactions between two individuals and result in an adaptive response in the receiver, here specifically copepodamides excreted by copepods [9]. Presently, 21 copepodamide compounds have been identified and their composition is copepod species specific [10]. *Pseudo-nitzschia seriata* increases DA production as a response to the naturally occurring mixture of copepods and to at least three isolated copepodamides in ecosystem-relevant doses [10, 11]. Cellular signal transduction depends on the perception of external cues, but how the diatoms or other phytoplankton species perceive the predator cues is still poorly understood. Perception by signal transduction via G protein-coupled receptors is proposed for the dinoflagellate *Alexandrium catenella* exposed to a heterotrophic dinoflagellate grazer [12]. When exposed to different grazers (copepods), the induced defense response and the discrimination between copepod species were suggested to be regulated by protein kinases and calcium signaling [13]. To our knowledge, only one other study has investigated the molecular mechanism behind grazer-induced defense mechanisms in a diatom. Gene regulation and metabolomic response in *Skeletonema marinoi* as a response to grazers showed that transcripts linked to G protein-coupled receptors and to nitric oxide synthesis were differentially expressed, and that *Skeletonema* reacts the same way in the presence of two different copepod species [14].

Several species of the diatom genera *Pseudo-nitzschia* and *Nitzschia* pose a threat to organisms in the marine environment due to their ability to produce DA, a marine biotoxin known to cause severe harm up the food web, and amnesic shellfish poisoning in humans. To date 52 *Pseudo*-*nitzschia* species are described and exactly half of these (26) plus two *Nitzschia* species have been reported to produce DA [15]. *Pseudo-nitzschia* was demonstrated to be a source of DA already in 1988, but the ecological role of toxin production is still not fully understood. *Pseudo-nitzschia* cells react to several abiotic and biotic environmental factors such as changes in light, temperature, salinity, pH, pCO_2_ and nutrient levels, as well as to the presence of bacteria and grazers by changing the production of toxins [16, 17].

As a result of a major research effort which began during the early 1990s, the biosynthetic pathway of DA is gradually being uncovered. Using ^13^C- and ^14^C-labelled precursors to detect the major metabolic pathways for DA synthesis, it was suggested that DA originates from condensation of a C10 isoprenoid with a tricarboxylic acid (a product of the citric acid cycle) [18, 19]. The precursor of the C10 isoprenoid was suggested to be geranyl pyrophosphate (GPP) and originate from acetyl CoA through the mevalonate (MEV) pathway [20] and the tricarboxylic acid proposedly derives from l-glutamate [18]. GPP is synthesized through the isopentenyl pyrophosphate pathway and originate either through (1) the proposed MEV pathway located in the cytosol and/or (2) an alternative route in the plastid, the methyl-erythritol phosphate metabolic (MEP) pathway [21] (Additional file 1: Figure S1). Both pathways are present in diatoms but the MEP pathway is not complete in all diatoms [21, 22]. The early findings have been confirmed and it was demonstrated that the amino function in DA is generated by nucleophilic displacement of GPP by an unknown intermediate [23]. Recently, six compounds with potential functions in the condensation of GPP and l-glutamate were identified and their structure described [24]. The same study also confirmed *N*-geranyl-l-glutamic acid as a potential intermediate. Four candidate genes, DabA-D, that can code for the condensation of GPP and L-glutamate towards DA were recently identified [25]. These four genes were upregulated under phosphate limitation or elevated pCO_2_, both conditions known to induce DA production [26]. DabA catalyzes the *N*-geranylation of L-glutamate to form *N*-geranyl-l-glutamic acid, while DabC and D catalyze dainic and isodomoic acids. The final isomerization reaction to complete the condensation to DA remains unknown. Another tricarboxylic acid, proline, a structural analog to DA, is suggested to be implicated in DA biosynthesis. Based on isotopic labeling pattern of DA, a coupling of DA production to the proline metabolism was suggested [18]. Proline could be an upstream precursor of DA, and a correlation between *Pseudo-nitzschia* cells with high accumulation of DA and low proline content has been found [27].

The principal aim of this study is (1) to acquire information on genes involved in the metabolic processes of DA synthesis in a species of *Pseudo-nitzschia*. For revealing whether the MEP or the MEV pathway is a part of the metabolic pathway, and whether we can confirm previous suggestions on genes involved in DA synthesis. (2) To acquire information on the molecular mechanism of perception of predator cues by studying transcriptional processes in the two diatoms *Pseudo-nitzschia* and *Fragilariopsis*. The target species tested are *P. seriata*, known to induce DA when exposed to predator cues, and a species of *Fragilariopsis* not known to induce this defense mechanism.

## Results

### Domoic acid content and growth rates

The cellular toxin content in *Pseudo*-*nitzschia* increased by sevenfold (t-test P = 0.032) as a response to predator cues exposure, while no increased toxin production was found in the controls (t-test P = 0.19). *Fragilariopsis* did not produce DA neither in response to grazer cues nor in the control. Both species grew exponentially and *Fragilariopsis* had a higher division rate than *Pseudo*-*nitzschia* (t-test P < 0.05) 0.64 ± 0.01 day^−1^ and 0.12 ± 0.06 day^−1^, respectively. (See details on DA levels and division rates in Table 1).

**Table 1:** Cell concentrations at the start of the experimental treatments, cellular growth rates and cellular domoic acid (DA) contents

Diatom treatment	# Cells start/end (cells mL^−1^)	Division rate (day^−1^)	Cellular DA start/end (pg DA cell^−1^)
*Pseudo*-*nitzschia*			
Predator cues	3947 ± 189/4952 ± 627	0.10 ± 0.06	0.1 ± 0.01/0.7 ± 0.2
Control	4245 ± 297/5924 ± 750	0.15 ± 0.05	0.1 ± 0.01/0.1 ± 0.02
*Fragilariopsis*			
Predator cues	2797 ± 38/6768 ± 156	0.63 ± 0.01	nd
Control	2771 ± 10/6644 ± 192	0.64 ± 0.01	nd

Number of replicates = 3. Results are given as mean and standard deviation, nd = no detection

### Overview of gene expression profiles in *Pseudo*-*nitzschia* and *Fragilariopsis* when exposed to predator cues

**Fig. 1:**
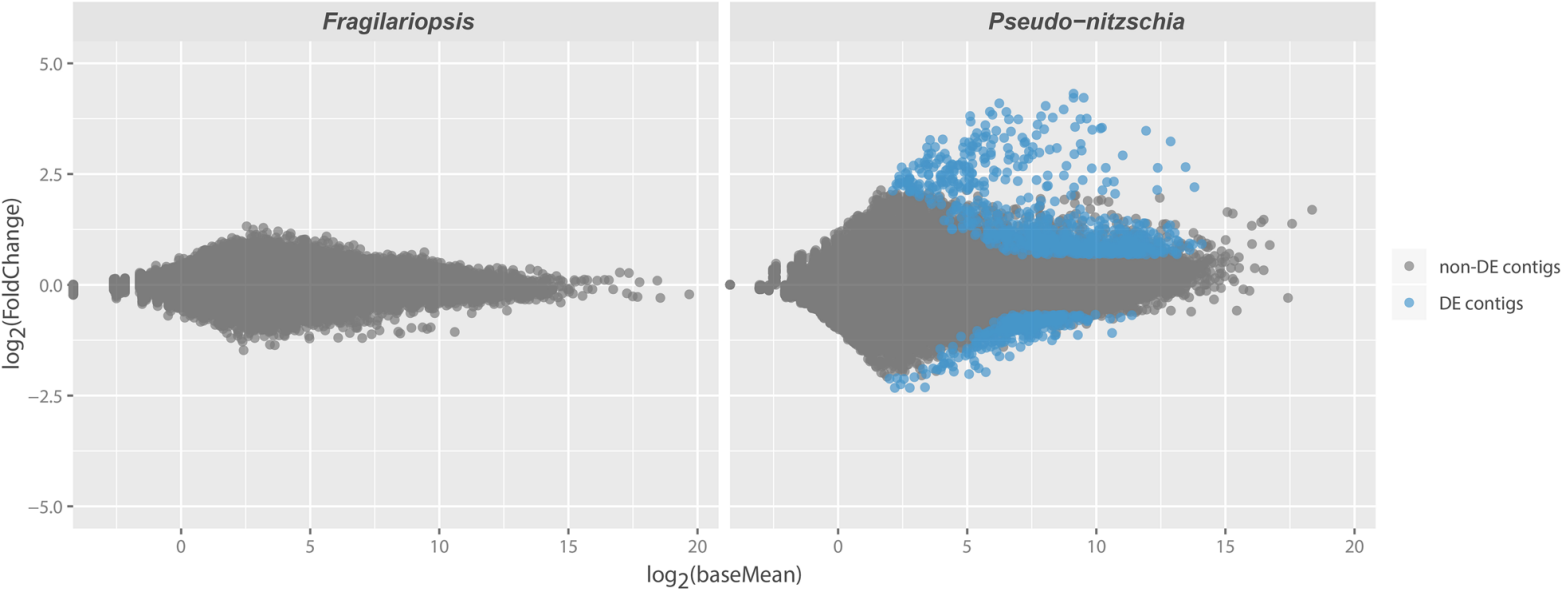
MA-plots for the gene expression data from *Fragilariopsis* and *Pseudo*-*nitzschia*. Both plots show the average expression of the normalized data on the x-axis and the log fold change ratios on the y-axis. Grey dots are non-differentially expressed genes (non-DE contigs) with a fold change below 1.5 and adjusted P-value above 0.05, number of replicates = 3. Blue dots are significantly differentially expressed genes (DE contigs) above the fold change threshold. Each dot represents a contig

Of the two diatoms, only *Pseudo*-*nitzschia* showed differential gene expression when exposed to predator cues (Fig. 1). In total 1128 genes were differentially expressed, of which 814 genes were upregulated and 314 genes downregulated compared to the control (Additional file 2: Tables S1 and S2). Based on the KEGG and KOG databases, 285 of the regulated genes (25% of the total) have a functional annotation. The remaining 75% of the regulated genes have a BLAST hit but could not be assigned to any specific function. These contigs are comparable to sequences in the data bases but their function is unknown. The assembly information is given in Table 2.

**Table 2:** Summary of RNAseq assemblies for *Pseudo*-*nitzschia* and *Fragilariopsis* number of replicates = 3

	*Pseudo*-*nitzschia*	*Fragilariopsis*
Number of reads	272.616.642	263.814.668
N 50	1759	1375
Number of contigs	66.423	71.590
Average contig length BP	811	788

**Fig. 2:**
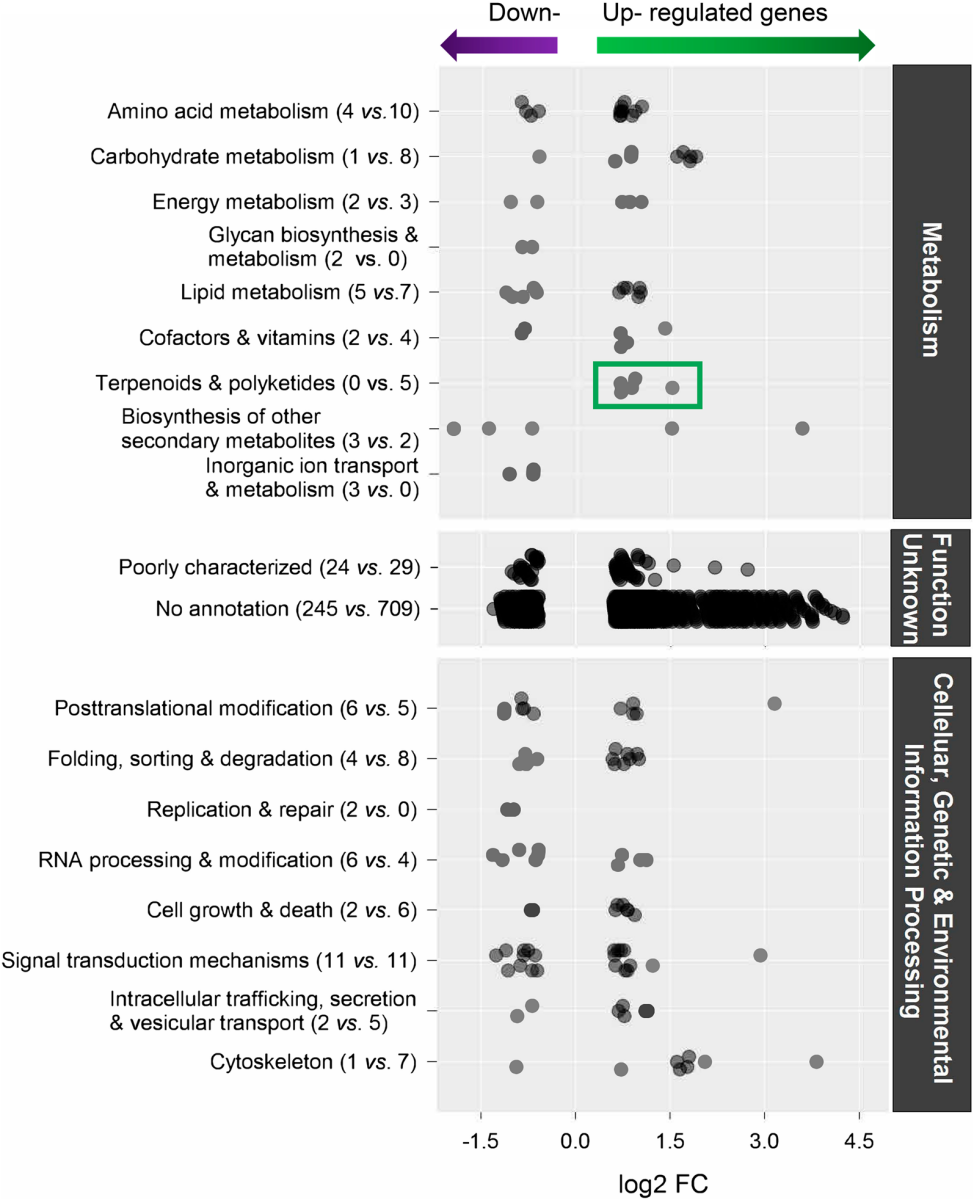
Breakdown of the major upregulated and downregulated genes in the KEGG and KOG database categories. The green box indicates candidate genes for the C10 isoprenoid in the biosynthesis of DA, number of replicates = 3

**Fig. 3:**
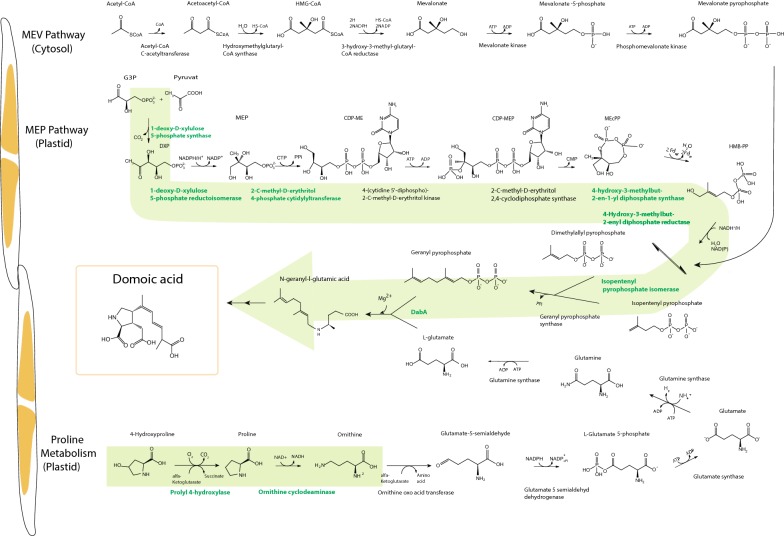
Illustration of the major metabolic pathways for the biosynthesis of domoic acid (DA). The precursor of the C10 isoprenoid can either arise via the mevalonate (MEV) pathway that is located in the cytosol and/or an alternative route in the plastid, the methyl-erythritol phosphate metabolic (MEP) pathway. The tricarboxylic acid is proposedly derived from l-glutamate. The green arrow indicates where genes are upregulated when *Pseudo*-*nitzschia* is triggered to produce DA, the assigned enzymes are highlighted bold green, number of replicates = 3. Mean fold change (FC) and adjusted P values as well EC numbers for the enzymes and are given in Table 6

Several genes involved in major metabolic pathways such as amino acid, carbohydrate and lipid metabolism are differentially expressed, with the majority (> 70%) of genes being upregulated compared to the control (Fig. 2). Differentially expressed genes assigned to the category of cellular information processing mainly showed a higher expression compared to the control. Genes were differentially regulated, both up and down, in the category of genetic information processing with functions in folding, sorting and degradation of RNA as well as replication and repair. In the category of environmental information processing, the 22 differentially expressed genes were equally either down- or upregulated (Fig. 2). Most importantly five upregulated genes have a function in the isopentenyl pyrophosphate pathway, assigned to the category of terpenoid and polyketide synthesis (highlighted green in Figs. 2 and 3), these are candidate genes for the C10 isoprenoid in the biosynthesis of DA. The majority of contigs could not be assigned to a function. All of the contigs with highest upregulation belong to this category.

### Signal transduction

We searched the transcripts for genes involved in cellular signal transduction, as the perception of external cues must transduce cascading molecular responses. A vesicular neurotransmitter transporter with a known function as extra cellular membrane interaction transporter (PSN0026807) is downregulated. Translation initiation factor 4E (PSN0006232) and serum/glucocorticoid-regulated kinase 2 (PSN0000383) are upregulated as well as GTP-binding protein (PSN0015960) and a RING-type E3 ubiquitin transferase (PSN00338470). Downstream regulator for G-binding proteins, mitogen-activated protein kinase (PSN0000026) and a mitogen-activated protein kinase homolog (PSN001617) are downregulated. The results indicate that *Pseudo*-*nitzschia* transduce signals from the copepods via G protein pathways. Nitric oxide signaling may be involved in the response to grazers and a nitric oxide synthase-interacting protein (PSN0002048) is upregulated. Genes in the category of signal transduction differentially expressed in *Pseudo*-*nitzschia* as response grazers are listed in Table 3.

**Table 3:** Differentially expressed gene involved in signal transduction

Signal transduction					
Accession number	Mean FC	Adjusted P values	Gene name	Description	Enzyme EC:
PSN0008622	7.64	< 0.001	NA	Leucine rich repeat	NA
PSN0015333	2.34	0.006	NA	Tyrosine kinase specific for activated GTP bound p21cdc42Hs	NA
PSN0007679	1.82	0.01	NA	Natriuretic peptide receptor guanylate cyclase	NA
PSN0000026	1.77	0.025	NA	MEKK and related serine threonine protein kinases	NA
PSN0022790	1.73	0.047	NA	Predicted GTPase activating protein	NA
PSN0012156	1.71	< 0.001	FLS2	LRR receptor-like serine threonine-protein kinase	2.7.11.1
PSN0007370	1.65	0.027	NA	Tyrosine kinase specific for activated GTP bound p21cdc42Hs	NA
PSN0001687	1.62	0.002	NA	Tyrosine kinase specific for activated GTP bound p21cdc42Hs	4.3.1.12
PSN0015960	1.62	< 0.001	RRAGA B	GTP binding protein	
PSN0008559	1.58	0.017	CALM	Calmodulin and related proteins EF hand superfamily	NA
PSN0008776	1.53	0.013	NA	Tyrosine kinase specific for activated GTP bound p21cdc42Hs	NA
PSN0004431	1.53	0.024	NA	Ca2 + calmodulin dependent protein kinase EF hand protein superfamily	NA
PSN0026544	− 1.56	0.031	NA	Tyrosine kinase specific for activated GTP bound p21cdc42Hs	NA
PSN0014002	− 1.6	0.026	NA	Tyrosine kinase specific for activated GTP bound p21cdc42Hs	NA
PSN0009159	− 1.61	0.0183	NA	Uncharacterized conserved protein	NA
PSN0006134	− 1.68	< 0.001	NA	LRR receptor-like serine threonine-protein kinase	NA
PSN0008421	− 1.76	0.005	PDCD4	Neoplastic transformation suppressor Pdcd4 MA 3 contains MA3 domain	NA
PSN0012753	− 1.77	< 0.001	NA	Leucine-rich repeat receptor-like protein kinase	NA
PSN0018357	− 1.83	0.007	NA	CDK5 activator binding protein	NA
PSN0004142	− 1.9	< 0.001	NA	Endocytosis signaling protein EHD1	NA
PSN0016217	− 2.15	0.039	NA	Serine threonine specific protein phosphatase PP1 catalytic subunit	NA
PSN0000124	− 2.37	0.008	NA	Endocytosis signaling protein EHD1	NA

Mean fold change (FC) and adjusted P values are based on three replicates

### Candidate genes for stress

There is strong indication that *Pseudo*-*nitzschia* cells are under stress when exposed to grazers. Various genes known to be a response to stressors are upregulated. Heat shock proteins and transcription factors are among the highly upregulated genes, like the HSP90 and one of its homologs HSP90-7, HSP40, heat shock factor protein 2, heat stress transcription factor A-2, and cytochrome P450 genes. Genes indicating cellular stress in *Pseudo*-*nitzschia* as response to the presence of grazers are listed in Table 4.

**Table 4:** Differently expressed genes indicating cellular stress in *Pseudo*-*nitzschia* in presence of predator cues from *Calanus*

Cellular stress					
Accession number	Mean FC	Adjusted P value	Gene name	Description	Enzyme EC:
PSN0046491	3.76	0.004	HSF2	Heat shock factor protein 2	NA
PSN0008255	2.6	< 0.001	RALDH1/ALDH-E1	Retinal dehydrogenase/aldehyde dehydrogenase family 1	1.2.36
PSN0002391	1.7	0.001	DNAJB2	Homolog subfamily B member 2/Heat shock 40 kDa protein 3	NA
PSN0017080	1.63	0.042	HSF 1/HSTF 3A	Heat shock factor protein 1	NA
PSN0009322	1.56	0.016	OsHsf-18	Heat stress transcription factor-A-2b	NA
PSN0001244	1.5	0.044	HSP90B	Heat shock protein 90 kDa beta	NA
PSN0002436	− 1.6	0.037	CYPIVDS8	Cytochrome P450 4d8	NA
PSN0019585	− 2.03	0.003	NA	Glutathione *S*-transferase	NA

Mean fold change (FC) and adjusted P values are based on three replicates

### Common grazing responses in diatoms

In *Fragilariopsis*, not a single gene is differentially expressed as a response to grazers (Fig. 1). When comparing the regulated nucleotide sequences in *Pseudo*-*nitzschia* with a similar study conducted on *Skeletonema* [14] we found congruence in upregulated genes. We found 13 genes, (out of only 34 and 55 matches, depending on time point in *Skeletonema*) that match upregulated genes in *Skeletonema* (Additional file 1: Figure S2; Additional file 3: Tables S5 and S6). Unfortunately, all these 13 genes lack annotations, which makes it difficult to reconstruct mechanisms for the response (Table 5). However, 10 of these 13 genes seem to be functionally related i.e. they show high similarities at the nucleotide level (Additional file 1: Figure S3; Additional file 3: Table S9). The oxide synthase-interacting protein (PSN0002048) is upregulated, but this not a homolog to c9307_gl_il in [14] or MMMETSP104-20121108 1594 in [22].

**Table 5:** Differently expressed genes we suggest to be common diatom responses to grazers

Grazing					
Accession number	Mean FC	Adjusted P value	Gene name	Description	Enzyme EC:
PSN0003667	10.12	< 0.001	NA	NA	NA
PSN0003517	8.76	< 0.001	NA	Cell wall-associated hydrolase	NA
PSN0000079	6.23	0.007	NA	Cell wall-associated hydrolase	NA
PSN0000487	6.19	0.008	NA	NA	NA
PSN0001476	5.02	0.027	NA	NA	NA
PSN0008314	4.87	0.026	NA	NA	NA
PSN0001176	4.72	0.024	NA	NA	NA
PSN0000367	4.66	0.035	NA	NA	NA
PSN0000066	4.61	0.029	NA	Cell wall-associated hydrolase	NA
PSN0000080	4.5	0.021	NA	Cell wall-associated hydrolase	NA
PSN0001342	4.46	0.045	NA	NA	NA
PSN0000500	4.27	0.015	NA	NA	NA
PSN0005577	2.43	0.019	NA	NA	NA

Mean fold change (FC) and adjusted P values are based on three replicates

### Candidate genes for domoic acid biosynthesis

Based on evidence and indications in earlier studies of DA production in *Pseudo*-*nitzschia*, we searched specifically for genes involved in the regulation of the isopentenyl pyrophosphate pathway, such as acetyl-CoA, isopentenyl pyrophosphate (via both the MEV and MEP pathways), GPP biosynthesis, l-glutamate biosynthesis including proline degradation into l-glutamate, and the citric acid cycle. All genes involved in both pathways to isopentenyl pyrophosphate were confirmed to be present in the transcriptome of *Pseudo*-*nitzschia* and *Fragilariopsis*. Five out of seven genes coding for enzymes involved in the MEP pathway were upregulated under grazer-induced DA induction. The gene encoding for isopentenyl pyrophosphate isomerase responsible for the condensation into GPP was also upregulated, as was the DabA gene involved in the condensation of *N*-geranyl-l-glutamic acid (Table 6, Fig. 3). None of the six genes in the proposed MEV pathway were significantly differentially expressed (Fig. 3). The non-significant regulation of the genes in the MEV pathway is randomly up or downregulated. Genes directly involved in the synthesis of l-glutamate: glutamate dehydrogenase [EC: 6.3.1.2] and glutamate synthase [EC: 4.7.1; EC:1.4.13; EC:1.4.13] were not differentially expressed compared to the control. No gene regulation was detected in genes known to encode for enzymes in the citric acid cycle. But prolyl 4-hydroxylase and ornithine cyclodeaminase [EC: 1.14.11.2 and 4.3.1.12], directly involved in the formation of proline, were upregulated (Fig. 3, Table 6), indicating proline to be the downstream precursor for l-glutamate.

**Table 6:** Genes regulated in the domoic acid (DA) induced grazer treatments that are involved or suggested to be involved in the biosynthesis of DA

Domoic acid					
Accession number	Mean FC	Adjusted P value	Gene name	Description	Enzyme EC:
In the methylerythritol phosphate metabolic pathway (MEP) to geranyl pyrophosphate (GPP)					
PSN0016235	1.88	< 0.001	Dxr	1-Deoxy-d-xylulose 5-phosphate reductoisomerase	1.1.1.267
PSN0004050	1.82	< 0.001	ispH	4-Hydroxy-3-methylbut-2-enyl diphosphate reductase	1.17.1.2
PSN0005356	1.61	0.043	ispG	4-Hydroxy-3-methylbut-2-en-1-yl diphosphate synthase	1.17.7.1
PSN0002600	1.60	< 0.001	ispD	2-C-Methyl-d-erythritol 4-phosphate cytidylyltransferase, chloroplastic	2.7.7.60
PSN0023754	1.60	0.013	ispE	4-Diphosphocytidyl-2-C-methyl-d-erythritol kinase	2.7.1.148
In the synthesis from MEP to GPP and l-glutamate towards domoic acid					
PSN0015669	12.23	< 0.001	DabB	Hypothetical protein	NA
PSN0029114 + PSN002498	11.23	< 0.001	DabC	Dioxygenase	NA
PSN0007588	9.41	< 0.001	DabA	*N*-prenyltransferase	NA
PSN0005772	2.83	<0.001	idi1-2	Isopentenyl-diphosphate Delta-isomerase	5.3.3.2
PSN0020364	2.83	< 0.001	DabD	Cytochrome P450	NA
Proline metabolism					
PSN0000830	1.67	< 0.001	P4HA	Prolyl 4-hydroxylase	1.14.11.2
PSN0001687	1.62	0.002	NA	Ornithine cyclodeaminase	4.3.1.12

Mean fold change (FC) and adjusted P values are based on three replicates

### Comparison with other studies on the biosynthetic pathway in *Pseudo*-*nitzschia* by gene expression analysis

Gene expression in *Pseudo*-*nitzschia multiserie*s at low and high DA production induced by silicate limitation has been investigated [28]. When comparing exponential vs. stationary phase, the study suggests 12 genes to be involved in DA production. Ten of these are present in *Pseudo*-*nitzschia seriata* but were not differentially expressed at induced DA conditions. By investigating three *Pseudo*-*nitzschia* species, one of which is toxigenic, *Pseudo*-*nitzschia multistriata*, one gene involved in nitrite oxide synthesis was confirmed to be present only in the DA-producing strain [22]. Neither this gene sequence nor any other hit for a nitric oxide synthesis enzyme was present in *P. seriata* in our study. Gene expression in *P. multistriata* was investigated under two DA-inducing conditions, phosphate limitation and elevated pCO_2_ [25]. We looked for regulation of genes involved in either of the isoprenoid pathways within the *P. multistriata* data set available on JGI, but did not find direct evidence for either pathway. One gene upregulated under all three conditions (grazer exposure, phosphate limitation and elevated pCO_2_) is PSN000450, which encodes for an enzyme in the MEP pathway (Table 6, Fig. 3). All four (DabA-D) genes from the [25] study are upregulated in *P. seriata* (Table 6, Fig. 3) and are not present in *Fragilariopsis*.

Among the 1028 regulated nuclear contigs in *P. seriata*, 441 match the 788 protein sequences in *P. multistriata.* Out of those, 23 are regulated in *P. seriata* exposed to grazers, and in *P. multistriata* under phosphate limitation, while nine are also differentially expressed under pCO_2_ elevation. Thus, nine genes are differentially regulated in all three DA-inducing conditions, eight of which are upregulated and one downregulated (Additional file 1: Figure S2; Additional file 3: Tables S3 and S4).

## Discussion

### Diatom response to grazers

We report here > 1000 genes differentially regulated in *Pseudo*-*nitzschia* as a response to copepod grazers while no differential gene expression was detected in *Fragilariopsis* under the same conditions. The two phylogenetically closely related phytoplankton species have therefore evolved different response strategies to threats from grazers. The lack of response in gene expression levels in *Fragilariopsis* indicates that in this taxon, protection against grazing might have evolved as a constitutive resistance mechanism, a trait that is always present. Hence, the results indicate that *Fragilariopsis* has not developed a sensory system to detect the predator cues from *C. finmarchicus* in the way shown here for *Pseudo*-*nitzschia* and for *Skeletonema* [14]. *Fragilariopsis* species are known to be fast growing and able to adapt to various types of habitat, and they successfully prevail in polar regions, both in the pelagic and in sea ice, as well as being globally distributed [29, 30]. A thick silica frustule as in *Fragilariopsis,* opposed to a thinner frustule in *Pseudo*-*nitzschia*, can provide mechanical protection—an armor that the copepod either cannot break or, if consumed, may pass undamaged through the gut [31]. The lack of response at the gene expression level does not indicate grazer defense to be an inducible trait, but does not exclude that other *Fragilariopsis* species may modify frustule thickness on demand. Inducible defenses in diatoms triggered by presence of copepods are still scarcely studied. To date this trait has been documented only in *Skeletonema* and *Pseudo*-*nitzschia*. We compared our results with the available data demonstrating gene expression in *Skeletonema* as a response to the same grazer species, *C. finmarchicus,* at two time points [14, 31]. There are only 34 (time point 1) and 55 (time point 2) matches with the contigs from our *Pseudo*-*nitzschia* datasets, thus highlighting the genetic differences in the defense traits of the two diatoms (chain length versus toxin production). However, 13 of the genes are upregulated in *Pseudo*-*nitzschia* and *Skeletonema* at both time points. This strongly suggests those genes to be candidates for common grazer responses in diatoms.

The response of *Pseudo*-*nitzschia* to the predator cues, i.e. the induced DA production, supports previous findings [6–8, 10, 32]. The induction of toxins as a response to predator cues implies that DA is an induced defense mechanism. However, the defense role is still speculative as DA-containing cells are ingested by their predators independent of their toxicity. Phycotoxins may, however, be effective after ingestion. Thus, mortality has been detected in copepods after feeding on toxic *Pseudo*-*nitzschia* [8], and reduced escape response has been seen in copepods having fed on toxic *Pseudo*-*nitzschia* [33]. No cost of DA production is detected in this study in terms of reduction in the growth rate of *Pseudo*-*nitzschia* and the same has been seen in [6, 7, 32]. Reduction of growth rate correlated, however, with high DA production in [8]. One of the prerequisites for inducible defenses is that they save the organism energy, but reports of costs in relation to induced defense are few and this aspect needs further attention. High costs of plasticity may over time be eradicated by evolution [34, 35] and low costs (e.g. minor reduction in growth rate) can go undetected in laboratory experiments where factors such as nutrients and energy (light) are not limited [36]. Small differences may not be statistically significant but may have a strong ecological impact when scaled up to natural populations [37]. Consequently, growth as a physiological and metabolic prime parameter to estimate cost and the physiological status of the cells, might not be a sufficient way to detect the metabolic investment of unicellular organisms. Regulation of genes in the category cell growth and death were upregulated, indicating that the *Pseudo*-*nitzschia* cells are forced to relocate energy, possibly because of the allocation costs of the DA production. Cellular regulative processes and changes in metabolic pathways mirror metabolic costs in terms of energy equivalents such as consumption of ATP and NADPH. These are for example needed for the synthesis of new enzymes and metabolites. Various genes of the KEGG and KOG categories might be involved in costs of production of amino acids and carbohydrate metabolism that are essential for all protein biosynthesis, such as the provision of glucose for cellular respiration.

### The role of MEP in the biosynthesis of domoic acid

Given that DA is synthesized by GPP and l-glutamate, the results of the present study reveal that the MEP pathway is a very likely precursor pathway for synthesis from GPP and further to DA. The majority of the genes in this pathway are upregulated in *Pseudo*-*nitzschia* when cells are triggered to produce DA compared to the control. The presence of both the MEP and the MEV pathways has been identified in various toxigenic and non-toxic *Pseudo*-*nitzschia* species, and in one *Fragilariopsis* species [22]. All genes involved in both pathways were confirmed to be present in the cDNA library of *P. seriata* and *Fragilariopsis*. However, we did not detect any differential gene expression of genes coding for enzymes in the MEV pathway as a response to predator cues. The MEP pathway was relatively recently fully described [38, 39] and has the same central role as the MEV pathway, to form isoprenoids. Isoprenoids are a class of organic compounds essential for all plants, for forming sterols, brassinosteroids, cytokinins, phytols, plant hormones and carotenoids [21]. The two pathways are located in different compartments of the cell, the MEV pathway in the cytosol and the MEP pathway in the plastid. Our results thus suggest that the formation of DA occurs in the plastid.

We did not detect any regulation of genes involved in the processes of the citric cycle for the proposed precursor of l-glutamate. The only regulation detected that can give hints to the downstream precursors are two genes in the proline metabolism. This supports the hypothesis that proline is coupled with the synthesis of DA. In theory, proline can be metabolized to l-glutamate as illustrated on Fig. 3. However, proline is a stress-related amino acid in many plants and algae [40–42] and may be regulated for other purposes than DA synthesis.

## Conclusions

We propose that the C10 isoprenoid product for biosynthesis of DA arises from the plastid MEP pathway rather than the cytosolic MEV pathway, as five out of seven genes involved in the MEP pathway are upregulated in *Pseudo-nitzschia* cells triggered to produce DA. We did not detect any gene-regulation that can be traced to the proposed l-glutamate from the citric acid cycle as precursor for DA, but suggest that the l-glutamate precursor could derive from the proline metabolism. Further, we demonstrate that the two phylogenetically closely related species have evolutionary distinctly different strategies for coping with grazer threats and only *Pseudo*-*nitzschia* responds with an induced defense when exposed to predator cues. Finally, we suggest 13 genes with unknown function to be involved in the responses of diatoms to grazers.

## Methods

### Organisms and experimental set up

The diatom strains were established from samples collected in Disko Bay, Greenland and permission for sampling was issued by The Government of Greenland, Naalakkersuisut. *Pseudo*-*nitzschia seriata* (strain Disko 8) was isolated, June 2013; *Fragilariopsis* sp. (strain A4–14) was isolated, May 2014. Biovolume of Disko 8 cells is 1695 µm^3^ and of A4–14 is ~ 830 µm^3^. The strain of *P. seriata* Disko 8 has previously been shown to produce DA in the exponential growth phase as a response to predator cues from *Calanus finmarchicus* as well as to isolated compounds of predator cues, a group of polar lipids named copepodamides [9]. Both strains were cultured at the University of Copenhagen in L-medium [43] at 4 °C and a light:dark cycle of 16:8 m^−2^ s^−1^. Approximately 3 weeks prior to the experiments, the cells were cultured in L-medium with additional NH_4_ to avoid differences in concentrations among treatments due to NH_4_ excreted by the copepods [44]. *Calanus finmarchicu*s, originally collected from the Trondheim fjord and kept in culture at Biotrix Trondheim was used as source of predator cues. Diatom cells at a concentration of ~ 4000 cells mL^−1^ were placed into two-chambered incubators; the chambers were 720 mL each and connected via two apertures of 7.5 cm in diameter. The apertures were covered with a 5-μm mesh, which separated the organisms but allowed the predator cues to pass through [7]. The incubators were placed on a plankton wheel which rotated at 2 rounds m^−1^ for 24 h. They were sampled for cellular toxin content, diatom cell concentration and gene expression analysis using a 100-mL syringe. After a 24-h acclimation period to the experimental conditions, six copepods were placed in one of the chambers to produce predator cues during the experiment. The control was without exposure to animals. *Calanus finmarchicus* produces copepodamides both when grazing and when starving [8]. Prior to the experiment, the copepods starved for 24 h so that the gut content was cleared. To ensure a constant supply of predator cues during the experiment, the animals were feeding on *Pseudo*-*nitzschia* or *Fragilariopsis* in the adjacent chamber, respectively.

The experiment was conducted in a temperature controlled room at 4 °C with light intensity of ~ 100 µmol photons m^−2^ s^−1^ and a light:dark cycle of 16:8. The experiment was initially carried out in quadruplicates, but the analyses was conducted in triplicates (for experimental workflow see Additional file 1: Figure S4). After 3 days, the experiment was terminated.

### Diatom concentration and toxin measurements

After sampling for RNA extractions (below), 2 mL were sampled for diatom cell concentration and fixed in acidic Lugol’s solution. The cells were counted in a Sedgewick-Rafter chamber using an inverted light microscope (Olympus CKX31 at a 100× magnification), with a minimum of 400 cells.

The growth rate (*µ*) was determined using an exponential model:

1 $$\mu = \frac{{LnC_{t2 } {-}LnC_{t1 } }}{t2 - t1}$$

*C* is the cell concentrations at *t*_*1*_ start and *t*_*2*_ end of the experiment.

Division rate per day was calculated as:

2 $$Div.day^{ - 1 } = \frac{\mu }{LN(2)}$$

*µ* is the growth rate from Eq. 1.

For DA measurements 10 mL samples were transferred to glass tubes and centrifuged for 5 min at 4000*g*, supernatants were discarded and pellets transferred to 2 mL centrifugation tubes (Eppendorf, Hamburg, Germany) and frozen at − 20 °C. DA was measured using liquid chromatography coupled with tandem mass spectrometry following [45]. In brief, samples were measured on a Sciex API 4000QTrap hybrid triple quadrupole-linear ion trap mass spectrometer (Sciex, Darmstadt, Germany) coupled to a LC 1100 liquid chromatograph (Agilent, Waldbronn, Germany). Separation was performed on a reverse-phase chromatography on a C8 phase (50 × 2 mm, 3 μm Hypersil BDS 120 Å (Phenomenex, Aschaffenburg, Germany) at 20 °C. The flow rate was 0.2 mL min^−1^ and gradient elution was performed with two eluants, wherein eluant A was water and B was acetonitrile/water (95:5 v/v), and both contained 2.0 mM ammonium formate and 50 mM formic acid. DA was detected in the positive mode by using the quantitative mass transition *m/z* 312 > 266 and the qualitative transition *m/z* 312 > 161. An external four point calibration curve (10, 50, 100 and 500 pg µL^−1^) was used for calibration and the detection limit (S/N = 3) was determined as 0.7 pg µL^−1^.

### Harvesting and RNA extraction

After 3 days of incubation with predator cues, the incubators were taken off the wheel. The incubators were carefully turned around minimum 10 times to ensure equal mixing of the culture prior to sampling. From each container, 60 mL were poured into sterile 4 × 15 mL sterile centrifugation tubes (Sigma-Aldrich) and centrifuged at 4 °C and 3300*g* for 10 min (Algera X-12R, Beckam Coulter, USA). Resulting cell pellets were pooled and immediately mixed with 1 mL 60 °C hot TriReagent (Sigma-Aldrich, Steinheim, Germany) and transferred to a 2 mL sterile centrifugation tube containing acid washed glass beads. The cells were lysed using a tissue lyser (Power lyser 24, Qiagen, Hilden, Germany) at maximum speed for 45 s and immediately afterwards frozen in liquid nitrogen and stored at − 80 °C until further use.

### RNA-isolation

The cell lysate was thawed on ice and 200 µL of pure chloroform was added to each vial and vortexed for 15 s. The mixture was incubated for 5 min at room temperature, shaken and incubated again for 5 min, and afterwards centrifuged for 15 min at 4 °C with 12,000*g*. The upper aqueous phase was transferred to a new vial, 2 µL of 5 M linear acrylamide (10–20 µg mL^−1^) and 1/10 volume of 3 M sodium-acetate (pH 5,2–5,5) was added, the mixture was shaken and 1:1 of 100% isopropanol was added. The mixture was then vortexed and incubated overnight (up to 14 h) at − 20 °C to precipitate RNA. The RNA-pellet was collected by 20 min centrifugation at 4 °C and 12,000*g*. The liquid was carefully removed and the pellet was washed twice, first with 1 mL 70% aqueous EtOH, centrifuged for 10 min at 4 °C and 12,000*g* and afterwards with 96% EtOH and centrifuged for 5 min. After removing as much of the EtOH as possible, the pellet was left to air dry for 5–10 min. Finally, the pellet was dissolved in 20 µL RNA/DNA RNase free water (Qiagen, Hilden, Germany). The amount of the RNA was checked on a Qubit 2.0^®^ fluorometer (Life Technologies). A RNA purity check was performed using a NanoDrop ND-100 spectrometer (PeqLab, Erlangen, Germany). The integrity of the RNA was examined using the Nano Chip Assay with the 2100 Bioanalyzer device (Agilent Technologies, Böblingen, Germany). High quality RNAs, OD 260/280 > 2 and OD260/230 > 1.8, with intact ribosomal peaks (obtained from the Bioanalyzer readings) were used for building cDNA libraries.

### cDNA libraries

Using the reagents provided in the TruSeq Stranded Total RNA LibraryPrep Kit High Throughput, Illumina^®^ and following the provided manual, the polyA containing mRNA molecules were purified and fragmented. The RNA was reverse transcribed into double stranded cDNA library by fragmenting and using primers with random hexamers into first strand cDNA by reverse transcriptase. The RNA template was then removed and a replacement strand synthesized, incorporating dUTP in place of dTTP to generate double stranded (ds) cDNA. To prepare the ds cDNA for hybridization onto a flow cell, a single adenylate nucleotide was added to the 3′ ends of the blunt fragments to prevent them from ligating to one another during the adapter ligation reaction. A corresponding single thymine nucleotide on the 3′ end of the adapter provides a complementary overhang for ligating the adapter to the fragment. For ligating multiple indexing adapters to the ends of the ds cDNA RNA Adapter plated provided with the Illumina^®^ TruSeq^®^ kit. After ligation, the ds cDNA was amplified with PCR. The cDNA library quality was checked on an Agilent DNA 1000 Chip Assay with the 2100 Bioanalyzer device (Agilent Technologies, Böblingen, Germany).

Each replicate was indexed prior to sequencing, so all replicates were demultiplexed and mapped separately against the reference assembly High-throughput RNA sequencing was performed on a NextSeq Illumina at the AWI.

### RNAseq analysis

Using the CLC Genomics Workbench 10 (Qiagen), a de novo assembly was produced from the RNASeq reads obtained for each species. One replicate of each species failed, hence the RNAseq analysis was carried out in triplicates. The annotation of the assembled contigs was done by BLASTx search against the Clusters of Orthologous Groups of proteins, database (KOG) (downloaded 2015.02 from ftp://ftp.ncbi.nih.gov/pub/) [46] and the SwissProt database (release 2016.08) with an e-value cut-off of < 10e^−05^. The Kyoto Encyclopedia of Genes and Genomes (KEGG) orthology identifiers (http://www.kegg.jp/) were mapped from SwissProt identifiers at http://www.uniprot.org. The alignment of the RNAseq reads to the obtained contigs was done with the CLC Genomics Workbench 10 (Qiagen), with the large gap read mapping option and default settings. Differential expression analysis was done with DESeq2 in R [47, 48]. Genes were considered to be differentially expressed when adjusted P-values were less than 0.05 and the calculated fold changes between the control and the treatment exceeded 1.5. Annotations of differentially expressed contigs according to the KOG and KEGG system were merged and manually grouped into aggregated categories.

### Comparative transcript analysis

We compared our dataset with two recently published, thematically close studies [14, 25]: (1) The response to grazing copepods in a non-toxic diatom *Skeletonema marioni* was transcriptionally characterized [14]. (2) Four genes involved in the biosynthesis of domoic acid were identified by analyzing transcriptomic data under two growth conditions known to induce DA production in *Pseudo*-*nitzschia multiseries* [25]. The comparison of both datasets with our data was performed on the level of sequence similarities by BLAST searches. For the comparison with the Amato dataset, we used a nucleotide BLAST (BLASTn) search with a cut-off of e-25, and for the comparison with the Brunson dataset, we used a translated nucleotide query (BLASTx search) with a cut-off of e-50. Both cut-offs are conservative, but this increases the likelihood to find true orthologous genes in the comparative analysis.

## Additional files

**Additional file 1: Figure S1.** An overview of the proposed cellular metabolic pathways for domoic acid. **Figure S2.** Venn diagram showing a comparison of differently expressed genes in *Pseudo-nitzschia seriata* inducing domoic acid (DA) production in response to grazers with data from *P. multistriata* producing DA during phosphate limitation and pCO_2_ elevation and to *Skeletonema marinoi* exposed to copepod grazers. **Figure S3.** Graphical overview of the sequence similarities of the genes commonly identified to respond to copepod grazing.

**Additional file 2: Table S1.** Results from the Deseq2 analyses. Annotations based on KEGG and KOG for *Pseudo-nitzschia seriata*. **Table S2**. Results from the Deseq2 analyses. Annotations based on KEGG and KOG for *Fragilariopsis* sp.

**Additional file 3: Table S3.** Differentially expressed contigs in *Pseudo-nitzschia seriata* induced with predator cues compared with *P. multistriata* under phosphate limiting condition in [25]. **Table S4.** Differentially expressed contigs in *Pseudo*-*nitzschia seriata* induced with predator cues compared with *P. multistriata* exposed to elevated pCO_2_ in [25]. **Table S5.** Differentially expressed contigs in *Pseudo-nitzschia seriata* compared with *Skeletonema marinoi* at time point 2 in [14] both specie are exposed to predator cues. **Table S6.** Differentially expressed contigs in *Pseudo-nitzschia seriata* compared with *Skeletonema marinoi* at time point 1 in [14] both specie are exposed to predator cues. **Table S7.** Results from the sequence comparison by blastx search of contigs from this study with the gene sequences from [25]. **Table S8.** Results from the sequence comparison by blastn search of contigs from this study with the gene sequences from [14]. **Table S9.** Functional and structural annotation of the 13 candidate genes we suggest are involved in response to copepod grazers.

**Additional file 4.** Expression profile *of Pseudo-nitzschia seriata*.

**Additional file 5.** Expression profile of *Fragilariopsis* sp.

## Abbreviations

DA: domoic acid
GPP: geranyl pyrophosphate
MEV: mevalonate isoprenoid
MEP: methyl-erythritol phosphate-pathway

## References

[1] Calbet A (2001). Mesozooplankton grazing effect on primary production: a global comparative analysis in marine ecosystems. Limnol Oceanogr.

[2] Pančić M, Kiørboe T (2018). Phytoplankton defence mechanisms: traits and trade-offs. Biol Rev.

[3] Amin SA, Hmelo LR, van Tol HM, Durham BP, Carlson LT, Heal KR, Morales RL, Berthiaume CT, Parker MS, Djunaedi B (2015). Interaction and signalling between a cosmopolitan phytoplankton and associated bacteria. Nature.

[4] Basu S, Patil S, Mapleson D, Russo MT, Vitale L, Fevola C, Maumus F, Casotti R, Mock T, Caccamo M (2017). Finding a partner in the ocean: molecular and evolutionary bases of the response to sexual cues in a planktonic diatom. New Phytol.

[5] Bergkvist J, Thor P, Jakobsen HH, Wangberg SA, Selander E (2012). Grazer-induced chain length plasticity reduces grazing risk in a marine diatom. Limnol Oceanogr.

[6] Harðardóttir S, Pančić M, Tammilehto A, Krock B, Møller EF, Nielsen TG, Lundholm N (2015). Dangerous relations in the arctic marine food web: interactions between toxin producing *Pseudo*-*nitzschia* diatoms and *Calanus* copepodites. Mar Drugs.

[7] Tammilehto A, Nielsen TG, Krock B, Møller EF, Lundholm N (2015). Induction of domoic acid production in the toxic diatom *Pseudo*-*nitzschia seriata* by calanoid copepods. Aquat Toxicol.

[8] Lundholm N, Krock B, John U, Skov J, Cheng J, Pančić M, Wohlrab S, Rigby K, Nielsen TG, Selander E (2018). Induction of domoic acid production in diatoms—types of grazers and diatoms are important. Harmful Algae.

[9] Selander E, Kubanek J, Hamberg M, Andersson MX, Cervin G, Pavia H (2015). Predator lipids induce paralytic shellfish toxins in bloom-forming algae. Proc Natl Acad Sci.

[10] Grebner W, Berglund EC, Berggren F, Eklund J, Harðadóttir S, Andersson MX, Selander E (2018). Induction of defensive traits in marine plankton—new copepodamide structures. Limnol Oceanogr.

[11] Selander E, Berglund EC, Engström P, Berggren F, Eklund J, Harðardóttir S, Lundholm N, Grebner W, Andersson MX. Copepods drive large-scale trait mediated effects in marine plankton. In: Science Advanced; 2019. 10.1126/sciadv.aat5096 PMC6382395 30801004

[12] Wohlrab S, Tillmann U, Cembella A, John U (2016). Trait changes induced by species interactions in two phenotypically distinct strains of a marine dinoflagellate. ISME J.

[13] Wohlrab S, Iversen MH, John U (2010). A molecular and co-evolutionary context for grazer induced toxin production in *Alexandrium tamarense*. PLoS ONE.

[14] Amato A, Sabatino V, Nylund GM, Bergkvist J, Basu S, Andersson MX, Sanges R, Godhe A, Kiorboe T, Selander E (2018). Grazer-induced transcriptomic and metabolomic response of the chain-forming diatom *Skeletonema marinoi*. ISME J.

[15] Lundholm N. Taxonomic reference list of harmful micro algae. http://www.marinespecies.org/hab. Accessed 17 Jan 2019.

[16] Lelong A, Hégaret H, Soudant P, Bates SS (2012). *Pseudo*-*nitzschia* (Bacillariophyceae) species, domoic acid and amnesic shellfish poisoning: revisiting previous paradigms. Phycologia.

[17] Bates SS, Hubbard KA, Lundholm N, Montresor M, Leaw CP (2018). *Pseudo*-*nitzschia*, *Nitzschia*, and domoic acid: new research since 2011. Harmful Algae.

[18] Douglas DJ, Ramsey UP, Walter JA, Wright JLC (1992). Biosynthesis of the neurotoxin domoic acid by the marine diatom *Nitzschia*-*pungens* forma *multiseries*, determined with c-13-labeled precursors and nuclear-magnetic-resonance. J Chem Soc Chem Commun.

[19] Ramsey UP, Douglas DJ, Walter JA, Wright JL (1998). Biosynthesis of domoic acid by the diatom *Pseudo*-*nitzschia* multiseries. Nat Toxins.

[20] Pan Y, Bates SS, Cembella AD (1998). Environmental stress and domoic acid production by *Pseudo-nitzschia*: a physiological perspective. Nat Toxins.

[21] Lohr M, Schwender J, Polle JE (2012). Isoprenoid biosynthesis in eukaryotic phototrophs: a spotlight on algae. Plant Sci.

[22] Di Dato V, Musacchia F, Petrosino G, Patil S, Montresor M, Sanges R, Ferrante MI (2015). Transcriptome sequencing of three *Pseudo*-*nitzschia* species reveals comparable gene sets and the presence of nitric oxide synthase genes in diatoms. Sci Rep.

[23] Savage TJ, Smith GJ, Clark AT, Saucedo PN (2012). Condensation of the isoprenoid and amino precursors in the biosynthesis of domoic acid. Toxicon.

[24] Maeno Y, Kotaki Y, Terada R, Cho Y, Konoki K, Yotsu-Yamashita M (2018). Six domoic acid related compounds from the red alga, *Chondria armata*, and domoic acid biosynthesis by the diatom, *Pseudo*-*nitzschia* multiseries. Sci Rep.

[25] Brunson JK, McKinnie SMK, Chekan JR, McCrow JP, Miles ZD, Bertrand EM, Bielinski VA, Luhavaya H, Obornik M, Smith GJ (2018). Biosynthesis of the neurotoxin domoic acid in a bloom-forming diatom. Science.

[26] Sun J, Hutchins DA, Feng Y, Seubert EL, Caron DA, Fu F-X (2011). Effects of changing pCO2 and phosphate availability on domoic acid production and physiology of the marine harmful bloom diatom *Pseudo-nitzschia* multiseries. Limnol Oceanogr.

[27] Smith GJ, Ladizinsky N, Miller PE (2000). Amino acid profiles in species and strains of *Pseudo*-*nitzschia* from Monterey Bay California: insights into the metabolic role(s) of domoic acid. Harmful Algal Blooms.

[28] Boissonneault KR, Henningsen BM, Bates SS, Robertson DL, Milton S, Pelletier J, Hogan DA, Housman DE (2013). Gene expression studies for the analysis of domoic acid production in the marine diatom *Pseudo*-*nitzschia multiseries*. BMC Mol Biol.

[29] Lundholm N, Hasle GR (2008). Are *Fragilariopsis cylindrus* and *Fragilariopsis nana* bipolar diatoms?—morphological and molecular analyses of two sympatric species. Nova Hedwig Beih.

[30] Kropuenske LR, Mills MM, van Dijken GL, Bailey S, Robinson DH, Welschmeyer NA, Arrigoa KR (2009). Photophysiology in two major Southern Ocean phytoplankton taxa: photoprotection in *Phaeocystis antarctica* and *Fragilariopsis cylindrus*. Limnol Oceanogr.

[31] Hamm CE, Merkel R, Springer O, Jurkojc P, Maier C, Prechtel K, Smetacek V (2003). Architecture and material properties of diatom shells provide effective mechanical protection. Nature.

[32] Harðardóttir S, Hjort DM, Wohlrab S, Krock B, John U, Nielsen TG, Lundholm N (2018). Trophic interactions, toxicokinetics, and detoxification processes in a domoic acid-producing diatom and two copepod species. Limnol Oceanogr.

[33] Harðardóttir S, Krock B, Wohlrab S, John U, Nielsen TG, Lundholm N (2018). Can domoic acid affect escape response in copepods?. Harmful algae.

[34] DeWitt TJ (1998). Costs and limits of phenotypic plasticity: tests with predator-induced morphology and life history in a freshwater snail. J Evol Biol.

[35] Van Kleunen M, Fischer M (2007). Progress in the detection of costs of phenotypic plasticity in plants. New Phytol.

[36] Van Donk E, Ianora A, Vos M (2011). Induced defences in marine and freshwater phytoplankton: a review. Hydrobiologia.

[37] Bell G, Collins S (2008). Adaptation, extinction and global change. Evol Appl.

[38] Eisenreich W, Bacher A, Arigoni D, Rohdich F (2004). Biosynthesis of isoprenoids via the non-mevalonate pathway. Cell Mol Life Sci.

[39] Lichtenthaler HK (2010) Chapter 7 The Non-mevalonate DOXP/MEP (Deoxyxylulose 5-Phosphate/Methylerythritol 4-Phosphate) Pathway of Chloroplast Isoprenoid and Pigment Biosynthesis. In: Rebeiz CA, Benning C, Bohnert HJ, Daniell H, Hoober JK, Lichtenthaler HK, Portis AR, Tripathy BC (eds) The Chloroplast. Advances in Photosynthesis and Respiration, vol 31. Springer, Dordrecht, pp 95–118.

[40] Rai V (2002). Role of amino acids in plant responses to stresses. Biol Plant.

[41] Krell A, Funck D, Plettner I, John U, Dieckmann G (2007). Regulation of proline metabolism under salt stress in the psychrophilic diatom *Fragilariopsis cylindrus* (bacillariophyceae). J Phycol.

[42] Rokitta SD, von Dassow P, Rost B, John U (2016). P-and N-depletion trigger similar cellular responses to promote senescence in eukaryotic phytoplankton. Front Mar Sci.

[43] Guillard RRL, Hargraves PE (1993). *Stichochrysis*-*immobilis* is a diatom, not a chyrsophyte. Phycologia.

[44] Corner E, Newell B (1967). On the nutrition and metabolism of zooplankton IV. The forms of nitrogen excreted by *Calanus*. J Mar Biol Assoc UK.

[45] Tammilehto A, Nielsen TG, Krock B, Møller EF, Lundholm N (2012). *Calanus* spp.—vectors for the biotoxin, domoic acid, in the Arctic marine ecosystem?. Harmful Algae.

[46] Tatusov RL, Fedorova ND, Jackson JD, Jacobs AR, Kiryutin B, Koonin EV, Krylov DM, Mazumder R, Mekhedov SL, Nikolskaya AN (2003). The COG database: an updated version includes eukaryotes. BMC Bioinform.

[47] Love MI, Huber W, Anders S (2014). Moderated estimation of fold change and dispersion for RNA-seq data with DESeq2. Genome Biol.

[48] Team RC (2013). R: a language and environment for statistical computing.

