# Supplementary material for: Transcriptomic responses to grazing reveal the metabolic pathway leading to the biosynthesis of domoic acid and highlight different defense strategies in diatoms
Source: BMC Mol Biol. 2019 Feb 26;20:7. doi: 10.1186/s12867-019-0124-0 (PMC6390554; doi:10.1186/s12867-019-0124-0)
Supplement: Supplementary file 1 — Additional file 1: Figure S1. An overview of the proposed cellular metabolic pathways for domoic acid. Figure S2. Venn diagram showing a comparison of differently expressed genes in Pseudo-nitzschia seriata inducing domoic acid (DA) production in response to grazers with data from P. multistriata producing DA during phosphate limitation and pCO2 elevation and to Skeletonema marinoi exposed to copepod grazers. Figure S3. Graphical overview of the sequence similarities of the genes commonly identified to respond to copepod grazing. [file 12867_2019_124_MOESM1_ESM.docx]

**Additional figures**

**Transcriptomic responses to grazing reveal the metabolic pathway leading to the biosynthesis of domoic acid and highlight different defense strategies in diatoms**

By Sara Harðardóttir, Sylke Wohlrab, Ditte Marie Hjort, Bernd Krock, Torkel Gissel Nielsen, Uwe John and Nina Lundholm

**Additional figures S1-S4**


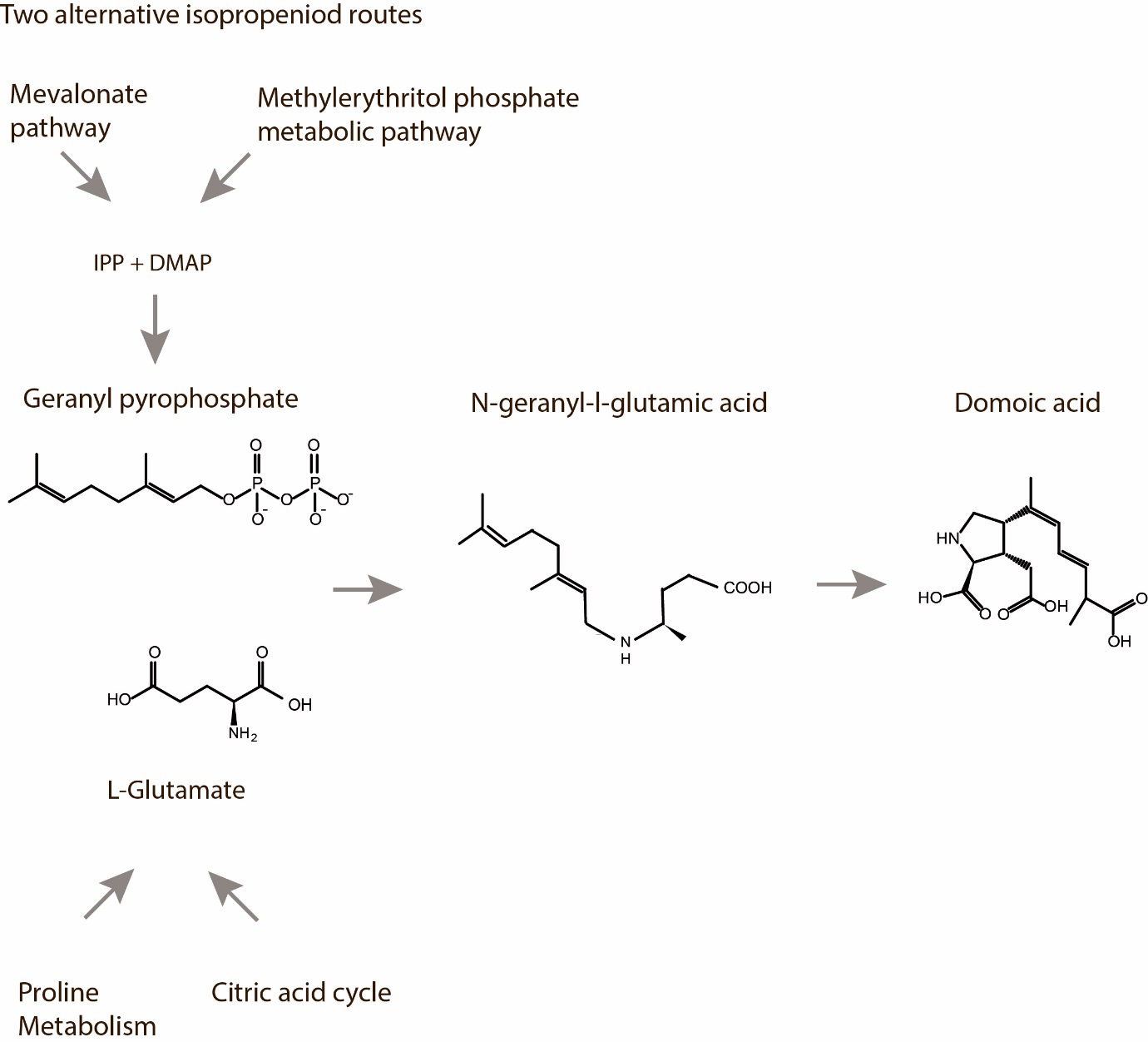
Figure S1. An overview of the proposed cellular metabolic pathways for domoic acidFigure S2. Venn diagram showing a comparison of differently expressed genes in *Pseudo-nitzschia seriata* inducing domoic acid (DA) production in response to grazers (this study) with data from *Pseudo-nitzschia multistriata* producing DA (Brunson S2; Brunson S3) and *Skeletonema marinoi* exposed to copepod grazers (Amato_T1, Amato_T2). Brunson_S2: *Pseudo-nitzschia multistriata* under phosphate limiting condition. Brunson S3*: P. multistriata* exposed to elevated pCO_2_. Amato T1 and T2 are two time points of *Skeletonema marinoi* exposed to grazer. The diagram illustrates that *P. seriata* inducing DA production in response to grazers has 23 genes in common only with *P. multistriata* under phosphate limiting condition. Nine of the differently expressed genes are the same in *P. seriata* and *P. multistriata* both inducing factors: Brunson S2; Brunson S3. Thirteen up regulated genes in *P. seriata* match upregulated genes in *Skeletonema* at both time points. Data underlying this graph can be found in the supplementary S3 table S3.1-6.


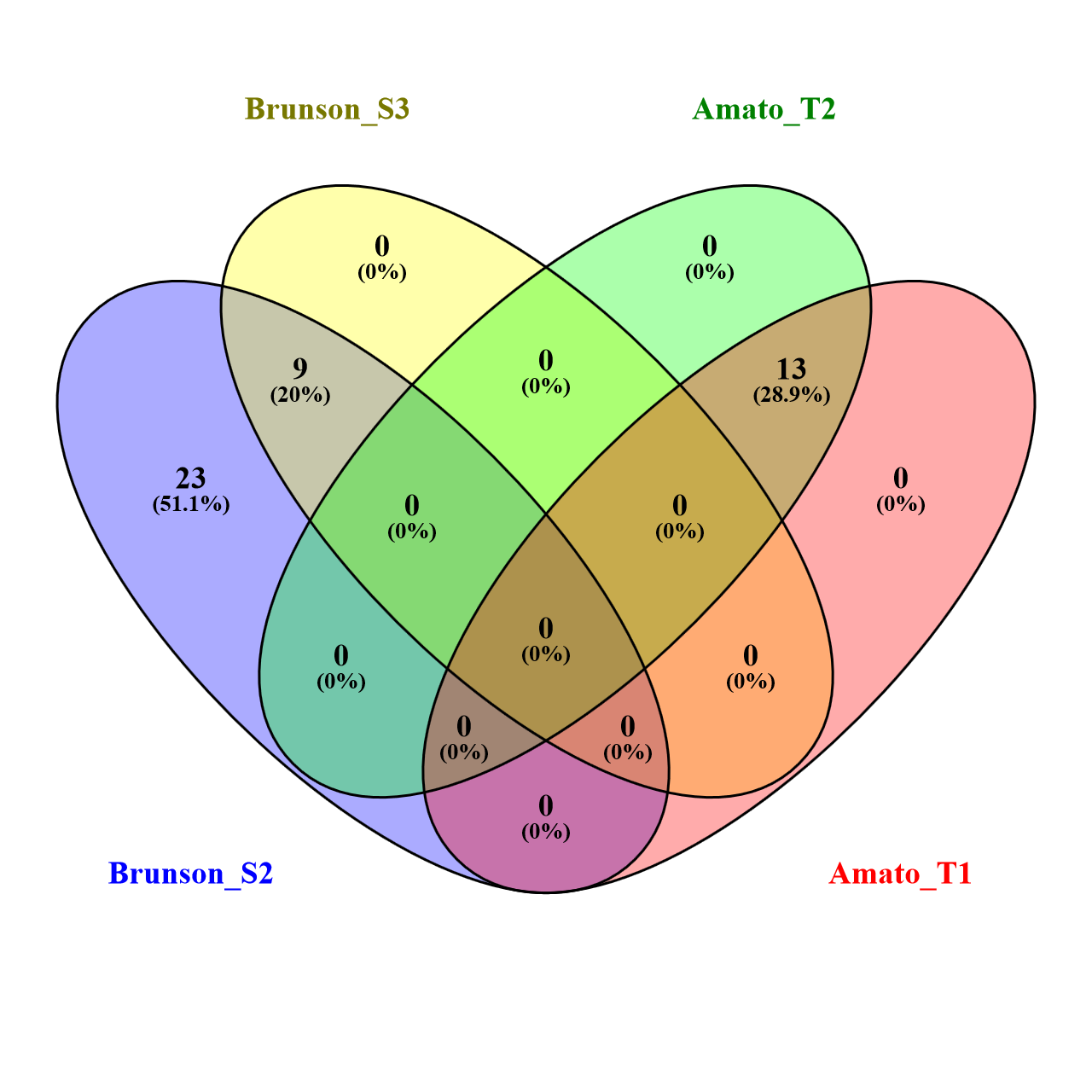


Amato A, Sabatino V, Nylund GM, Bergkvist J, Basu S, Andersson MX, Sanges R, Godhe A, Kiørboe T, Selander E, Ferrante MI. Grazer-induced transcriptomic and metabolomic response of the chain-forming diatom *Skeletonema marinoi*. The ISME journal. 2018;12(6):1594.

Brunson JK, McKinnie SM, Chekan JR, McCrow JP, Miles ZD, Bertrand EM, Bielinski VA, Luhavaya H, Oborník M, Smith GJ, Hutchins DA. Biosynthesis of the neurotoxin domoic acid in a bloom-forming diatom. Science. 2018;361(6409):1356-8.

Figure S3 Graphical overview of the sequence similarities of the genes commonly (this study and Amato et al.) identified to respond to copepod grazing. Each dot represents one of the 13 *Pseudo-nitzschia* contigs regulated as response to grazers, arrows with e-values give blastn similarity values of the two sequences (from reciprocal blast search). Data underlying this graph can be found in the supplementary S3 Table S3.7.


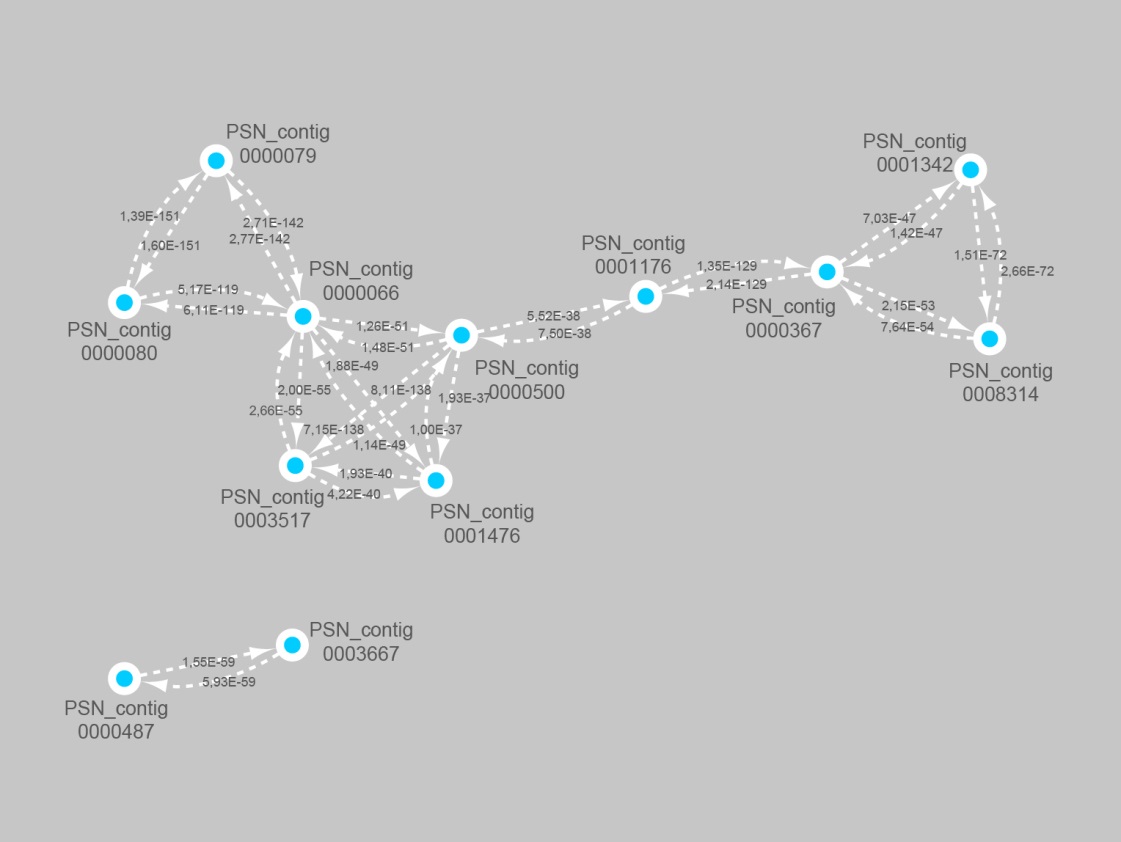


**Figure S4** Workflow of the experiment

**
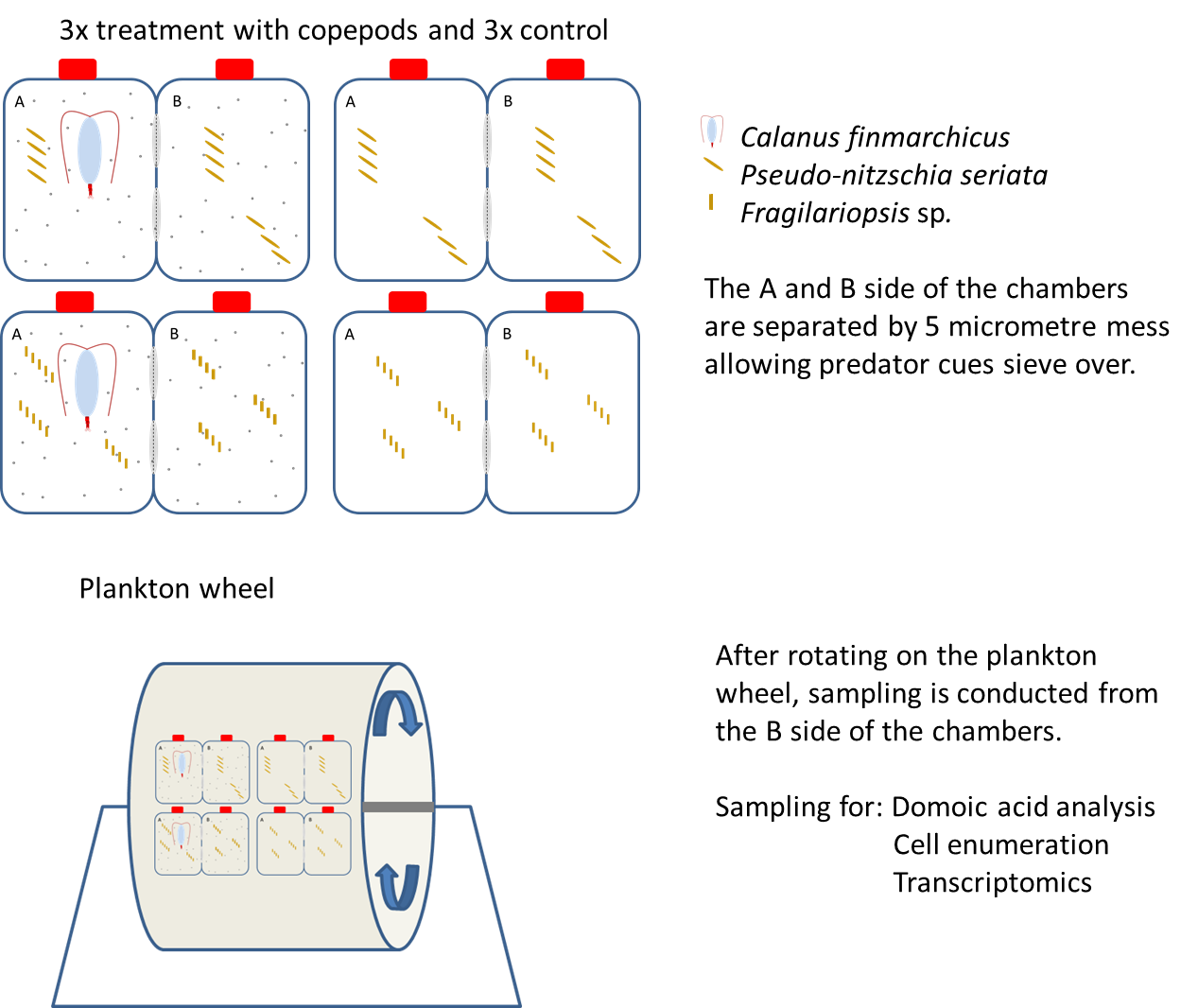
**
